# Phenylephrine Decreases Vascular Tension in Goat Arteries in Specific Circumstances

**DOI:** 10.1371/journal.pone.0158551

**Published:** 2016-06-30

**Authors:** Renu R. Raj, Sathya Subramani

**Affiliations:** Department of Physiology, Christian Medical College, Vellore, Tamil Nadu, India; Maastricht University, NETHERLANDS

## Abstract

Phenylephrine (PE) causes vasoconstriction through alpha adrenergic receptors. PE-induced vasodilatation has also been reported earlier in pre-constricted vessels. Here we demonstrate in spiral strips of goat arteries that addition of PE can decrease tone even from base-line levels (i.e. not pre-constricted) and show that this process requires nitric oxide (NO) and alpha adrenergic stimulation, but is cGMP-independent. Under control conditions, PE caused vasoconstriction, but under conditions where NO levels are higher, as with L-Arginine or sodium nitroprusside, PE decreased vessel tension. L-Arginine/PE combination was not able to decrease tension when alpha adrenoceptors were blocked with Phentolamine or endothelial nitric oxide synthase (eNOS) was blocked with Nω-Nitro-L-arginine (L-NNA). Propranolol, a beta blocker, was unable to prevent the reduction in tension by the L-Arginine/PE combination. Adrenaline and noradrenaline (and not isoproterenol) also reduced vessel tension in the presence of L-Arginine. Even when NO levels were not enhanced, relieving NO from having to stimulate the enzyme soluble guanylyl cyclase (sGC) (either by using sGC blockers, namely ODQ or methylene blue, or by enhancing cGMP levels (with sildenafil) which by negative feedback probably inhibits sGC) led to PE-induced reduction of vascular tension. PMA—phorbol myristate acetate—an agonist which stimulates Protein Kinase C was able to prevent the ability of PE to reduce vascular tension in a high NO environment. Our conclusion is that PE reduces vascular tension through alpha adrenoceptors if there is excess NO availability to activate a putative pathway. Though the reduction of vessel tone by PE is dependent on NO, it is independent of cGMP. Prior treatment with PMA or PE itself can prevent further PE-induced reduction of tension in a high NO environment. The results here suggest, counter-intuitively, that alpha blockers may be of help in the treatment of septic shock where nitric oxide levels are high.

## Introduction

Phenylephrine (PE) is an alpha adrenergic agonist, well known to induce vasoconstriction through Inositol triphosphate (IP_3_) mediated calcium release from sarcoplasmic reticulum [1]. However it has been reported that PE can cause vasodilatation through alpha 1D [2], alpha 2 [3] [4] or beta adrenergic receptor [5] mediated mechanisms. Specifically Filippi *et al* [2] report that while micro molar concentrations of PE produced contraction in rat mesenteric blood vessels with intact endothelium, nano molar concentrations caused vasodilatation in pre-constricted vessels. The relaxation was mediated through alpha 1D receptor and required nitric oxide (NO) [2]. Apart from these reports, to our knowledge, there are no further reports on vasodilatation occurring through alpha adrenergic stimulation.

Sympathetic vasodilatation itself is not a new concept and has been addressed for nearly a century now, reviewed by Joyner and Dietz [6]. Sympathetic cholinergic fibres were implicated as the cause for vasodilatation, but such sympathetic vasodilator fibres were not identified in humans. The vasodilatory mechanism under sympatho-excitation was however concluded to be NO-mediated.

NO was identified as the endothelium-derived relaxing factor and is a well-known vasodilator. It is produced from L-Arginine by the action of Nitric oxide synthase (NOS) enzyme. There are three isoforms, namely, eNOS, iNOS and nNOS of which eNOS is a constitutive enzyme present in the endothelium of blood vessels [7]. NO formed in the endothelium, diffuses into the vascular smooth muscle and is stated to cause vasodilatation by cGMP-dependent as well as independent pathways [8]. The cGMP-dependent mechanism involves activation of Protein Kinase G (PKG) and consequent activation of myosin light chain phosphatase. The cGMP-independent mechanism is suggested to be either reuptake of cytosolic calcium by SERCA [8] or activation of calcium-dependent potassium channels [9].

Here we report our observations on the effect of PE on changes in resting tension of spiral strips of small artery supplying skeletal muscle, isolated from goat legs. It is demonstrated that 10 μmol/L concentration of PE is sufficient to cause maximal vasoconstriction under control conditions, but the same and higher concentrations caused dose-dependent decrease in vessel tension under certain circumstances. Reduction of vessel tension by PE is mediated through alpha receptors and is NO-dependent, as was reported earlier [2]. Other alpha adrenergic agonists, namely adrenaline and noradrenaline also reduced vascular tension in the presence of L-Arginine, while the beta agonist Isoproterenol failed to reduce tension in similar circumstances. Propranolol, a beta blocker, failed to prevent the reduction in vessel tension produced by the L-Arginine/PE combination. Additionally, it is demonstrated here that the L-Arginine/PE combination reduces vessel tension in a cGMP-independent manner. The role of cGMP in PE-induced reduction of vessel tension is complex. PE was able to reduce vascular tension in combination with Sildenafil, which increases cGMP levels (by inhibition of the enzyme cGMP phosphodiesterase), and in combination with1H-[1,2,4] oxidiazolo [4,3-a]quinoxalin-1-one [ODQ, a soluble-guanylyl cyclase (sGC) enzyme inhibitor] which decreases cGMP levels. The reduction in tension that occurred either with PE/Sildenafil combination or with PE/ODQ combination was NO-dependent and did not occur in the presence of Nω-Nitro-L-arginine (L-NNA), a blocker of eNOS. An explanation is offered to explain the phenomenon. It is also suggested that the cGMP-independent, NO-dependent pathway of the PE-induced reduction of tension works through Protein Kinase C (PKC), because prior activation of PKC prevents the ability of PE to reduce vascular tension. Another important observation here is that base-line tension under control conditions remains low due to constitutive levels of NO and even this NO-induced basal relaxed state is cGMP-independent.

To our knowledge, this is the first report of the following phenomena: From the basal tone, tension can decrease further with a combination of PE and NO; maintenance of a low basal tone by NO, as well as reduction of tension by the combination of NO and PE are cGMP- independent; when the enzymes sGC or cGMP phosphodiesterase are inhibited, PE can reduce vascular tension.

The findings are significant as they suggest that use of alpha adrenergic agonists in conditions like septic shock can worsen the shock state and that alpha blockers may be of help. Methylene blue which is proposed in the treatment of septic shock can be detrimental, as it causes vasodilatation in combination with PE.

## Materials and Methods

Goat legs were obtained from a registered slaughter house (co-ordinates 12.93°N 79.13°E) close to the lab and were transported to the lab within 20 minutes of sacrifice in most cases, and not later than 45 minutes in any case. The legs were washed and the skin removed. A section of a small artery, about 2 cm in length, supplying skeletal muscle was isolated from the goat leg and cut spirally using a pair of fine scissors. One end of the spiral strip was tied to the base of an organ bath of 25 ml capacity, filled with physiological salt solution. The composition of the solution was as follows (in mmol/L): NaCl 100; KCl 3; CaCl_2_ 1.3; MgCl_2_ 2; Na_2_HPO_4_- 2; NaH_2_PO_4_−0.5; NaHCO_3_ 25; HEPES 10; Glucose 5, pH 7.4 with 1 molar NaOH. The solution was circulated through the inner chamber of the organ bath from a reservoir. Both the reservoir and the organ bath were double-walled, and temperature of the perfusate was maintained at 37°C by a circulating water bath which circulated water at that temperature through the outer jacket of the organ bath and reservoir. The solution was aerated with carbogen gas (95% oxygen and 5% carbon dioxide).

The other end of the strip was tied with a piece of silk thread to a force transducer which was connected to a computerized Data acquisition system (Power Lab). Optimal preload was applied to keep the thread taut. The resting tension with the preload was in the range 0.15 gm to 0.6 gm at the start of the experiment. Drugs were added to the organ bath and changes in tension in the spiral strip were recorded. The data was acquired at 1 KHz and was imported to Igor Pro (Wave metrics). The data was filtered using the Gaussian smoothening function in Igor Pro.

Histological examination of the spiral strip: Spiral strip was immersed in 10% buffered formalin for 2 days, then dehydrated with ascending grades of alcohol concentrations, and was cleared with xylene. Then the tissue was impregnated with liquid paraffin and paraffin block was made. Sections of 5 μm thickness were made using a microtome. Tissue sections were dewaxinated and hydrated with descending grades of alcohol and finally washed in distilled water. Tissue sections were stained with hematoxylin and eosin and were observed under light microscope.

Fluorescence staining with DAPI: Tissue sections were made as for light microscopy. After hydration, sections were washed with Phosphate buffered saline (PBS) and were stained with 4', 6-diamidino-2-phenylindole (DAPI) which is a nuclear stain. The tissue sections after staining were observed under fluorescence microscope.

All salts mentioned in the solution composition were purchased from SIGMA. L-Arginine, PE, Nω-Nitro-L-arginine (L-NNA),1H-[1,2,4] oxidiazolo [4,3-a]quinoxalin-1-one (ODQ),adrenaline, noradrenaline, isoproterenol, propranolol, phentolamine and phorbol 12- myristate 13- acetate (PMA) were also purchased from SIGMA.1000 times stock solutions of drugs were prepared and a suitable volume added to the bath to achieve final concentration. L-Arginine, PE, adrenaline, noradrenaline, phentolamine, propranolol and PMA stocks were made in distilled water. ODQ was dissolved in DMSO. L-NNA was dissolved in 1 mol/L HCl and pH was adjusted to 7.4 with 1 mol/L NaOH. DAPI was purchased from Cell Signalling Technology, Chennai.

### Statistical analysis

Statistical analysis was done using Wilcoxon signed rank (WSR) test to compare vascular tension before and after an intervention. To compare changes in vascular tension due to two different interventions, done in different samples, Mann- Whitney U test was used. P ≤ 0.05 was considered as statistically significant.

## Results

### PE induces vasoconstriction under control conditions

**Fig 1:**
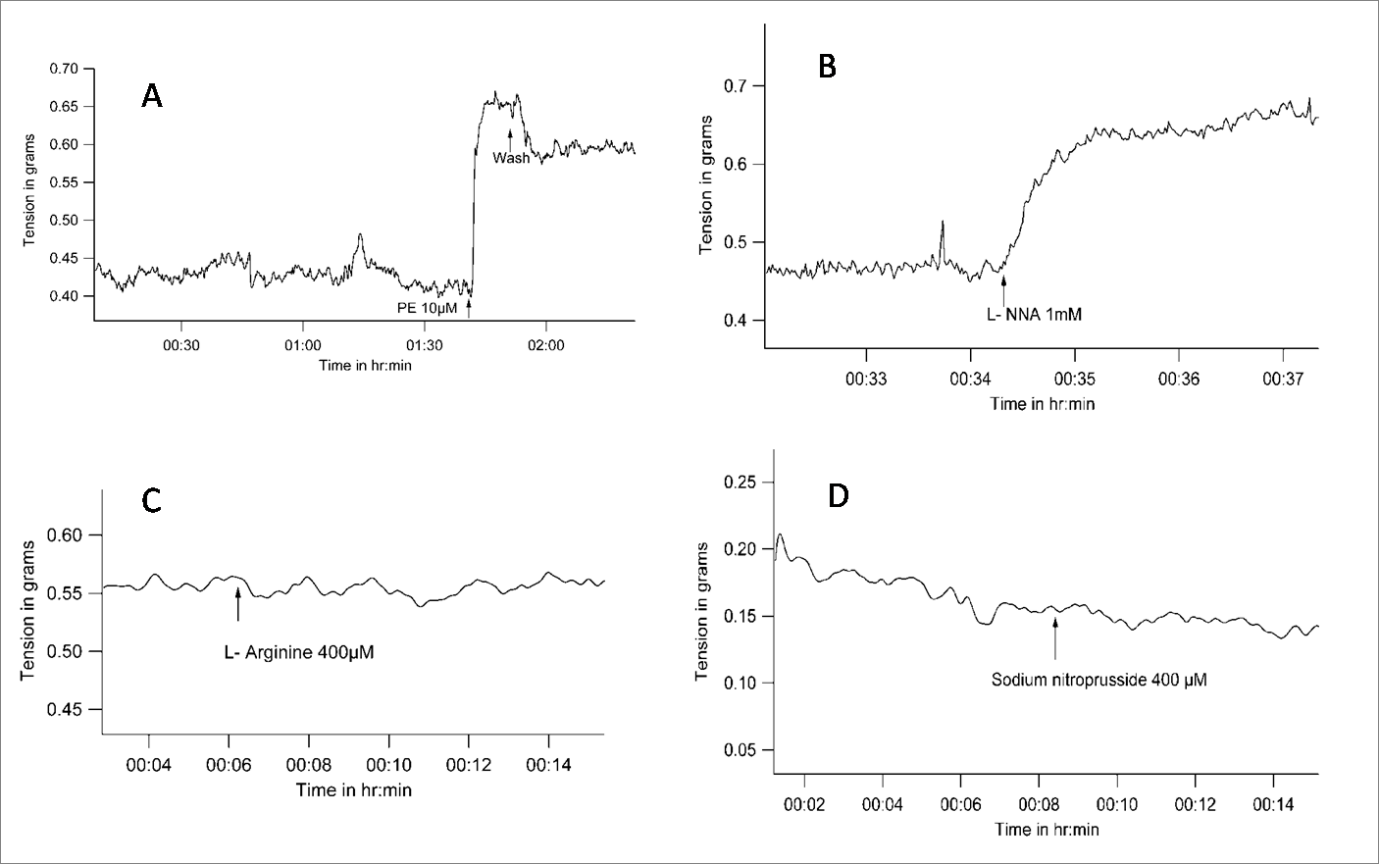
Representative tracings of tension recordings in spiral strips of small arteries from goat legs demonstrating *(A)*, vasoconstriction with Phenylephrine, *(B)*, vasoconstriction with L-NNA, *(C&D)*, lack of change in basal tone with nitric oxide donors L-Arginine and sodium nitroprusside respectively.

Under control conditions, PE increased the tone of the artery (Fig 1A). PE (10μmol/L) increased tension from 0.26 ± 0.12 gm to 0.41 ± 0.21 gm (mean ± SD, n = 5, P = 0.042 with WSR test when tensions before and after PE were compared).

### Constitutive synthesis of NO maintains a low basal tone; Additional NO does not have an effect on vascular tone by itself

When constitutive nitric oxide synthesis was blocked with L-NNA (1mmol/L), vessel tension increased from a base-line tension of 0.21 ± 0.15 gm to 0.43 ± 0.12gm (mean ± SD, n = 5, P = 0.043 with WSR test) (Fig 1B).

However, vessel tension did not decrease when nitric oxide levels were enhanced with L-Arginine (400μmol/L). The values (mean ± SD, n = 4) for vessel tension before and after L-Arginine were 0.42 ± 0.14 gm and 0.42 ± 0.13 gm (n = 5, P = 0.317with WSR test) (Fig 1C). Similarly, vessel tension did not change on addition of the nitric oxide donor SNP (400μmol/L), and remained at 0.28 ± 0.1 gm before and after SNP (n = 4, P = 0.317 with WSR test) (Fig 1D).

### PE reduces vascular tension in a high nitric oxide environment

**Fig 2:**
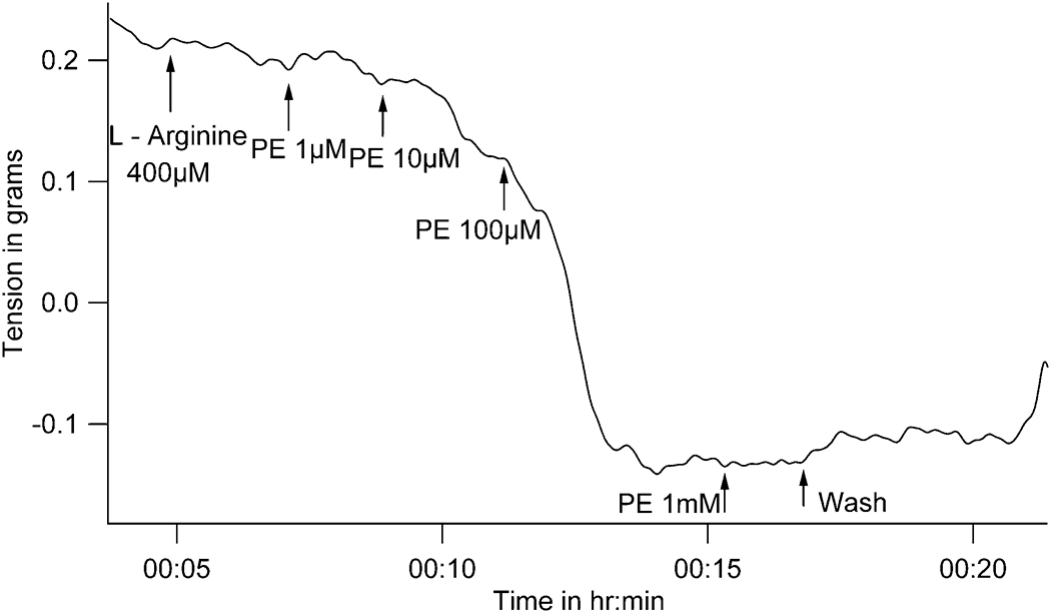
Representative tracing of tension recording in spiral strips of small arteries from goat legs demonstrating dose-dependent decrease in vascular tension by Phenylephrine in the presence of L- Arginine.

While L-Arginine did not change basal tone by itself, subsequent addition of PE reduced tone. In the presence of L- Arginine, PE was added in increasing doses from 1μmol/L to 1mmol/L. While 1 μmol/L PE did not change vessel tension, it decreased from 0.23 ± 0.06 gm to 0.11 ± 0.07 gm with 10μmol/L PE (n = 4, P = 0.058 with WSR test) and to 0 ± 0.12 gm with subsequent addition of 100μmol/L PE (P = 0.042 with WSR test) in the presence of L- Arginine. 1mmol/L concentration of PE did not cause any further decrease in vessel tension. Since statistically significant tension-reduction occurred with 100μmol/L PE, this dose was used in further experiments (Fig 2).

**Fig 3:**
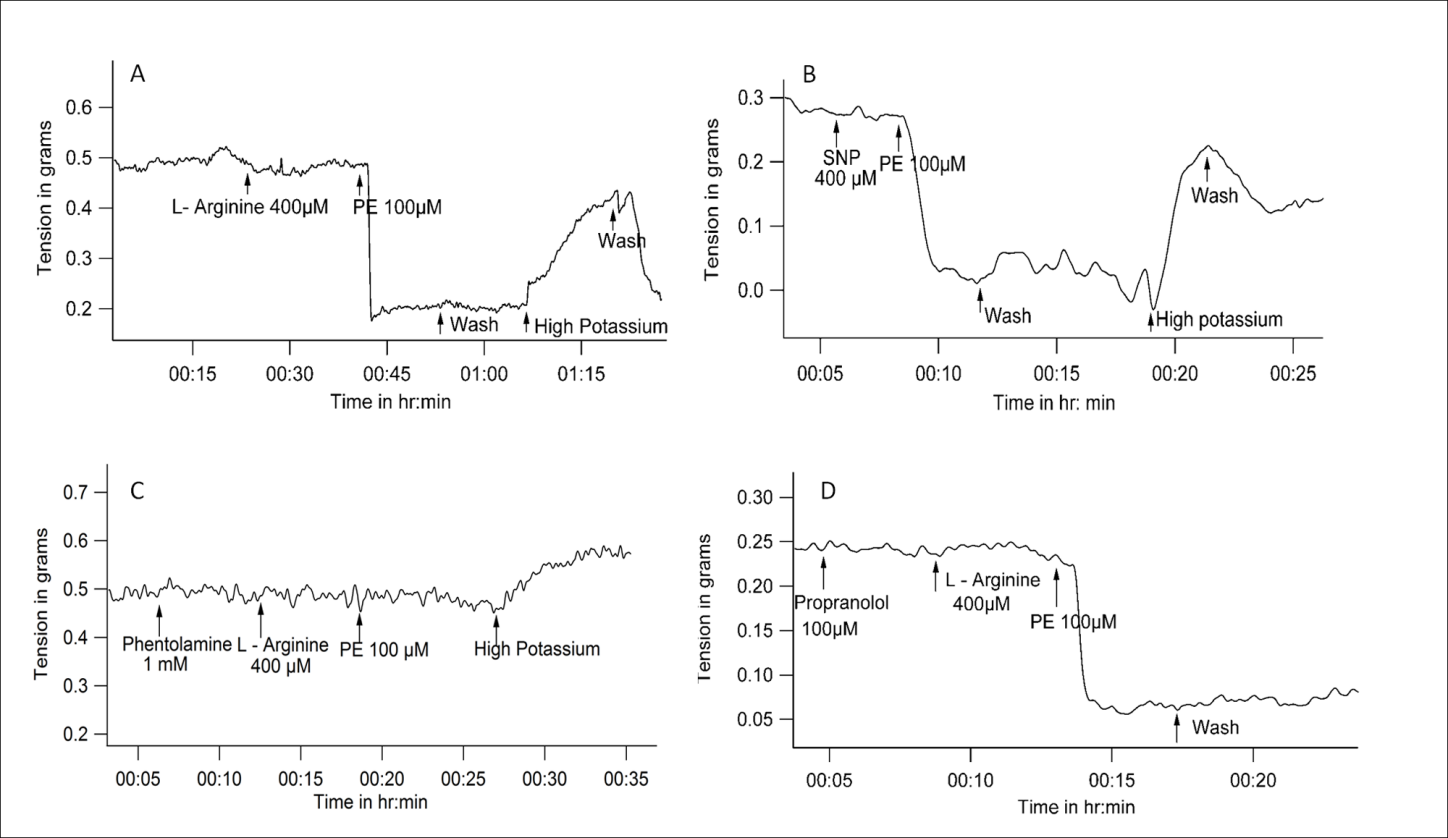
Representative tracings of tension recordings in spiral strips of small arteries from goat legs demonstrating *(A & B)*, Phenylephrine decreases vessel tension in the presence of L-Arginine and SNP, *(C)*, lack of reduction of vessel tension by Phenylephrine / L-Arginine combination in the presence of alpha adrenergic blocker phentolamine, *(D)*, reduction in vessel tension by Phenylephrine / L-Arginine combination in the presence of beta adrenergic blocker Propranolol.

In a separate set of experiments where only one concentration of PE was used, in the presence of L-Arginine, PE (100μmol/L) decreased vessel tension from 0.42 ± 0.13 gm to 0.24 ± 0.1gm (mean ± SD, n = 5, P = 0.043 with WSR test when tension before and after PE in the presence of L-Arginine were compared, Fig 3A). The reduction of tension in response to PE in the presence of L-Arginine was statistically significant when compared to the contractile response to PE alone (P = 0.009 with Mann- Whitney U test when percent changes in tension due to PE were compared between experiments where PE was used alone, and experiments where PE was used subsequent to L-Arginine).

Similarly in the presence of SNP, which can liberate nitric oxide, PE decreased vessel tension from 0.29 ± 0.1 gm to 0.13 ± 0.06 gm (mean ± SD, n = 5, P = 0.043 with WSR test when tension before and after PE in the presence of SNP were compared (Fig 3B).

### PE-induced reduction of tension in a high NO environment is due to specific alpha adrenergic receptor activation

Phentolamine is an alpha adrenergic blocker. When Phentolamine (1mmol/L) was present, the L-Arginine / PE combination could not decrease vessel tension (Fig 3C). Vessel tension did not change on addition of PE in the presence of phentolamine and L- Arginine, and remained at0.47 ± 0.05 gm before and after PE (P = 1 with WSR test). A high potassium solution was able to increase tone in the end, demonstrating viability of the preparation.

In the presence of L-Arginine, when the tension changes due to PE were expressed as a percentage of tension just before addition of PE, there was a statistically significant difference between the experimental groups where phentolamine was absent (59.75 ± 13.5%, mean ± SD, n = 5) or present (100%) (n = 5, P = 0.005 with Mann- Whitney U test).

Propranolol is a beta adrenergic blocker. Propranolol (100μmol/L) was unable to inhibit reduction of tension with L- Arginine/PE combination (Fig 3D). In the presence of Propranolol and L- Arginine, PE was still able to decrease vessel tension from 0.27 ± 0.09 gm to 0.12 ± 0.07 gm (n = 5, P = 0.042 with WSR test).

In the presence of L-Arginine, when the tension changes due to PE were expressed as a percentage of tension just before addition of PE, there was no statistically significant difference between the experimental groups where Propranolol was absent (59.75 ± 13.5%, mean ± SD, n = 5) or present (44.06 ± 17.8%) (n = 5, P = 0.117 with Mann- Whitney U test).

### Adrenaline and noradrenaline, (which are non-specific adrenergic agonists) also cause decrease in vascular tone in the presence of L-Arginine

**Fig 4:**
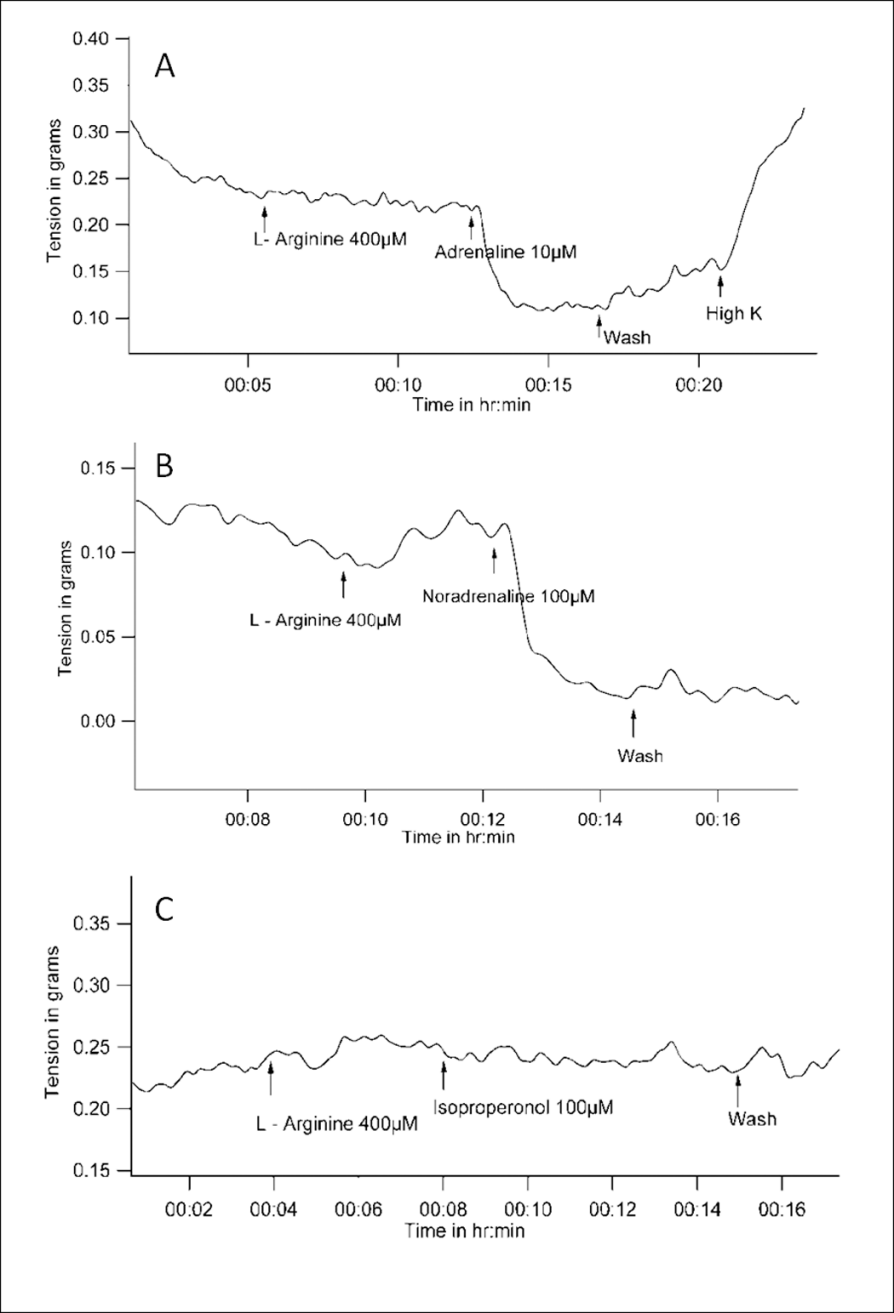
Representative tracings of tension recordings in spiral strips of small arteries from goat legs demonstrating *(A)* Reduction in vessel tension by L- Arginine/Adrenaline combination, *(B)*, Reduction in vessel tension by L- Arginine/Noradrenaline combination, *(C)*, Lack of change of vessel tension with isoproterenol / L- Arginine combination.

Adrenaline and noradrenaline are nonspecific adrenergic agonists which can activate both alpha and beta receptors. When adrenaline (10μmol/L) or noradrenaline (100μmol/L) was added subsequent to L-Arginine, there was reduction in vessel tension (Fig 4A and 4B). L- Arginine / adrenaline combination decreased vessel tension from 0.35 ± 0.1gm to 0.05 ± 0.1 gm (n = 5, P = 0.043 with WSR test). L-Arginine / noradrenaline combination decreased vessel tension from 0.23 ± 0.07 gm to 0.08 ± 0.04 gm (n = 5, P = 0.041with WSR test).

### Isoproterenol, (a beta adrenoceptor agonist, without effect on alpha receptors) does not cause reduction of tone in the presence of L-Arginine

Isoproterenol is a non-specific beta agonist. In an L- Arginine environment, isoproterenol (100μmol/L) did not change the tone of vessel. The vessel tensions before and after addition of isoproterenol (in the presence of L-Arginine) were 0.27 ± 0.05 gm and 0.28 ± 0.08 gm respectively. (n = 4, P = 0.317 with WSR test) (Fig 4C).

### Reduction of vascular tone due to L-arginine / PE combination is NO-dependent

**Fig 5:**
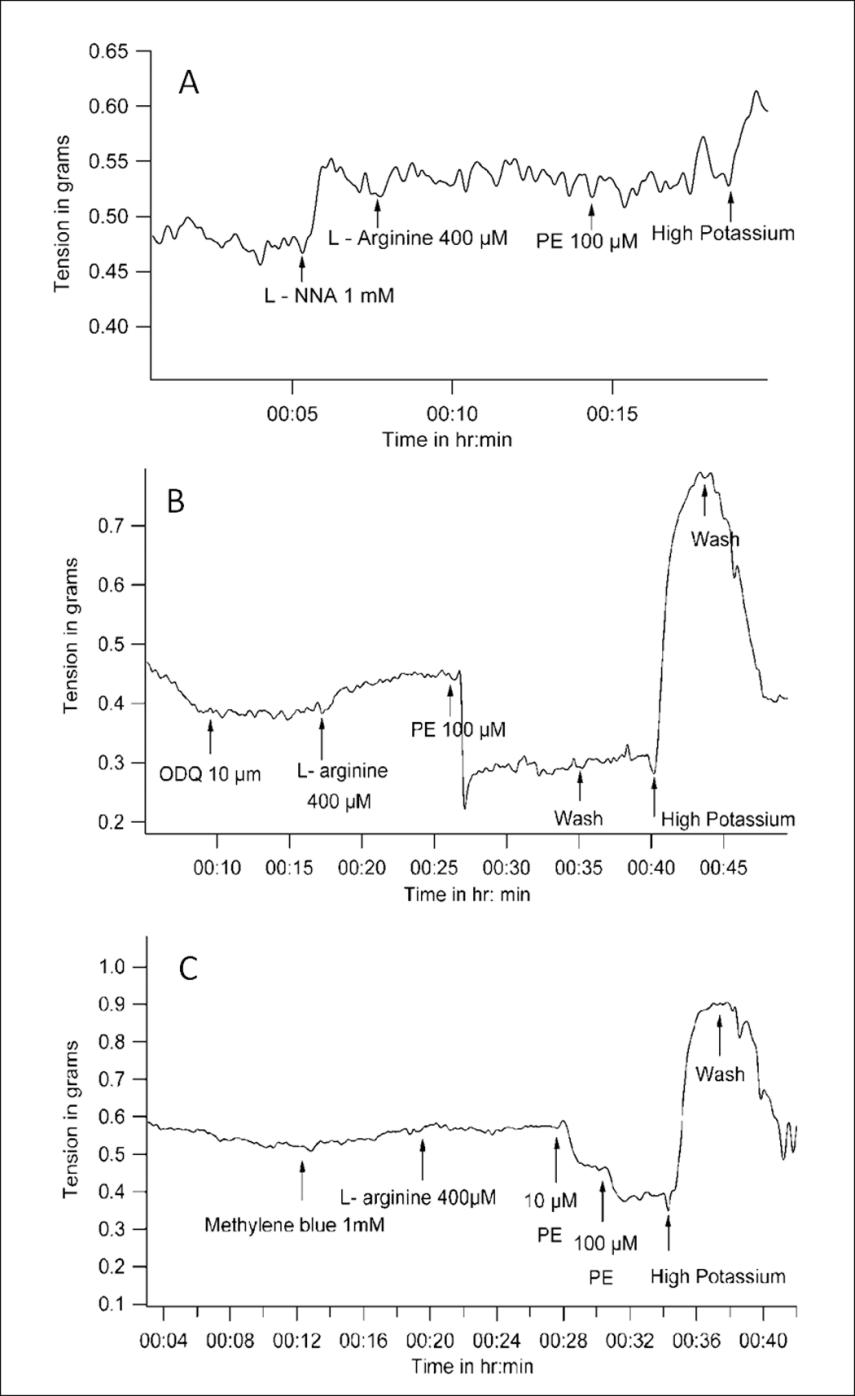
Representative tracings of tension recordings in spiral strips of small arteries from goat legs demonstrating*(A)*, lack of reduction of vessel tension by Phenylephrine / L-Arginine combination in the presence of eNOS inhibitor L- NNA, *(B & C)*, inability of sGC inhibitors ODQ and Methylene blue to prevent the reduction in vessel tension by Phenylephrine / L- Arginine combination.

L-NNA which is a competitive inhibitor of nitric oxide synthase, itself increased the basal tone of the artery as stated earlier. In the presence of L-NNA, L-Arginine / PE combination could not reduce tension. The value of vessel tension before and after PE in the presence of L- Arginine and L- NNA were 0.49 ± 0.12 gm and 0.5 ± 0.14 gm (n = 4, P = 0.18 with WSR test). In the presence of L-Arginine, when the tension changes due to PE were expressed as a percentage of tension just before addition of PE, there was a statistically significant difference between the experimental groups where L-NNA was absent (60 ± 14%, mean ± SD, n = 5) or present (103 ± 5%) (n = 4) (P = 0.014 with Mann- Whitney U test) (Fig 5A).

It must be noted that PE was not able to induce vasoconstriction either, in the presence of L-NNA. In the experiment shown in Fig 5A, potassium chloride (80 mmol/L) was used to depolarize the tissue in the end. The tissue was able to contract with high potassium, demonstrating that it was viable. Lack of relaxation response to L-Arginine and PE in the presence of L-NNA was therefore not due to death of tissue and was due to a specific lack of NO formation.

### Reduction of vascular tone that occurs with the L-Arginine/PE combination is cGMP-independent

ODQ is an inhibitor of sGC and thereby decreases cGMP levels. In the presence of ODQ (10 μmol/L), the L-Arginine / PE combination was still able to reduce vessel tension, causing it to decrease from 0.32 ± 0.16 gm (prior to PE) to 0.12 ± 0.11 gm, after addition of PE (n = 5, P = 0.042 with WSR test). In the presence of L-Arginine, when the tension changes due to PE were expressed as a percentage of tension just before addition of PE, there was no statistically significant difference between the experimental groups where ODQ was absent (59.75 ± 13.47%, mean ± SD, n = 5) or present (40.41 ± 17.53%) (P = 0.076 with Mann- Whitney U test). At the end of the experiment, high K^+^ solution was able to produce contraction, demonstrating that the tissue was viable and had the ability to contract (Fig 5B). Methylene blue (1mmol/L), another inhibitor of sGC also could not inhibit reduction in tension by the L-Arginine / PE combination. Vessel tension before and after PE in the presence of L- Arginine and methylene blue were 0.44 ± 0.11 gm and 0.29 ± 0.13 gm (n = 5, P = 0.043with WSR test). In the presence of L-Arginine, when the tension changes due to PE were expressed as a percentage of tension just before addition of PE, there was no statistically significant difference between the experimental groups where methylene blue was absent (59.75 ± 13.47%, mean ± SD, n = 5) or present (44.06 ± 17.8%) (n = 5) (P = 0.402 with Mann-Whitney U test) (Fig 5C).

### PE reduces vessel tension in the presence of sGC inhibitors or Sildenafil

**Fig 6:**
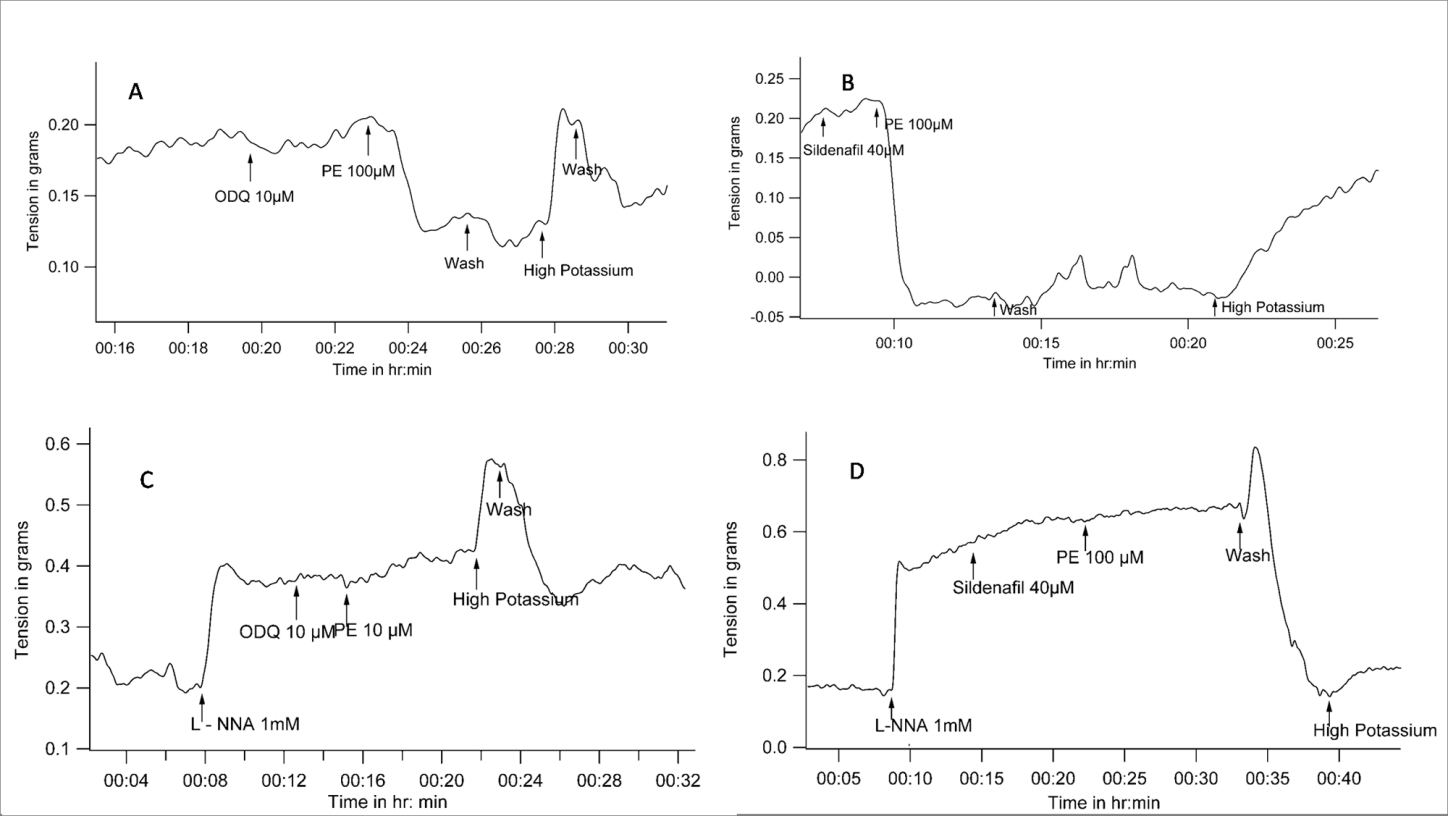
Representative tracings of tension recordings in spiral strips of small arteries from goat legs demonstrating that Phenylephrine can reduce tension in the presence of *(A)*, sGC inhibitor ODQ i.e., a condition where cGMP levels are expected to be low, *(B)*, cGMP phosphodiesterase inhibitor Sildenafil i.e., a condition where cGMP levels are expected to be high. However, Phenylephrine is unable to reduce tension with *(C)*, ODQ or *(D)*, Sildenafil when nitric oxide synthesis is blocked with L-NNA.

The sGC inhibitor ODQ by itself did not cause a change in basal tone. Vessel tension remained at 0.2 ± 0.09 gm before and after ODQ (n = 8, P = 0.18 with WSR test). Subsequent addition of PE caused reduction in tension. Vessel tensions before and after addition of PE in the presence of ODQ were 0.26 ± 0.1 gm and 0.08 ± 0.04 gm (n = 5, P = 0.043 with WSR test). PE-induced tension-reduction in the presence of ODQ was statistically significant when compared to the contractile response to PE alone (P = 0.009 with Mann-Whitney U test when percent changes in tension due to PE were compared between experiments where PE was used alone, and experiments where PE was used subsequent to ODQ) (Fig 6A).

Sildenafil is a cGMP phosphodiesterase inhibitor and is expected to produce vasodilatation by increasing cGMP levels. Sildenafil (40μmol/L) itself did not produce any change in the basal tone. Vessel tensions before and after sildenafil were 0.24 ± 0.1 gm and 0.2 ± 0.1 gm respectively (n = 5, P = 0.06 with WSR test). However, subsequent addition of PE caused the tension to decrease from 0.28 ± 0.1gm to 0.12 ±0.11 gm (n = 5, P = 0.043 with WSR test). Reduction in tension in response to PE in the presence of sildenafil was statistically significant when compared to the contractile response to PE alone (P = 0.009 with Mann-Whitney U test when percent changes in tension due to PE were compared between experiments where PE was used alone, and experiments where PE was used subsequent to sildenafil) (Fig 6B).

### PE-induced reduction of tension in the presence of sGC inhibitors or Sildenafil is also NO-dependent

In the presence of L-NNA, there was no relaxation with the ODQ / PE combination. The value of vessel tension before and after PE in the presence of ODQ and L- NNA were 0.33 ± 0.06 gm and 0.35 ± 0.06 gm (n = 4, P = 0.157 with WSR test). In the presence of ODQ, when the tension changes due to PE were expressed as a percentage of tension just before addition of PE, there was a statistically significant difference between the experimental groups where L-NNA was absent (34.03 ± 26.93%, mean ± SD, n = 5) or present (103.6 ± 3.12%) (n = 4) (P = 0.014 with Mann-Whitney U test) (Fig 6C).

Sildenafil / PE combination too did not cause relaxation in the presence of L-NNA. The value of vessel tension before and after PE in the presence of sildenafil and L- NNA were 0.53 ± 0.09 gm and 0.55 ± 0.08 gm (n = 4, P = 0.18 with WSR test). In the presence of sildenafil, when the tension changes due to PE were expressed as a percentage of tension just before addition of PE, there was a statistically significant difference between the experimental groups where L-NNA was absent (34.03 ± 26.93%, mean ± SD, n = 5) or present (104.1 ± 3.77%) (n = 4) (P = 0.018 with Mann-Whitney U test) (Fig 6D).

### Protein Kinase C activation prevents vasodilatation due to L-Arginine / PE combination

**Fig 7:**
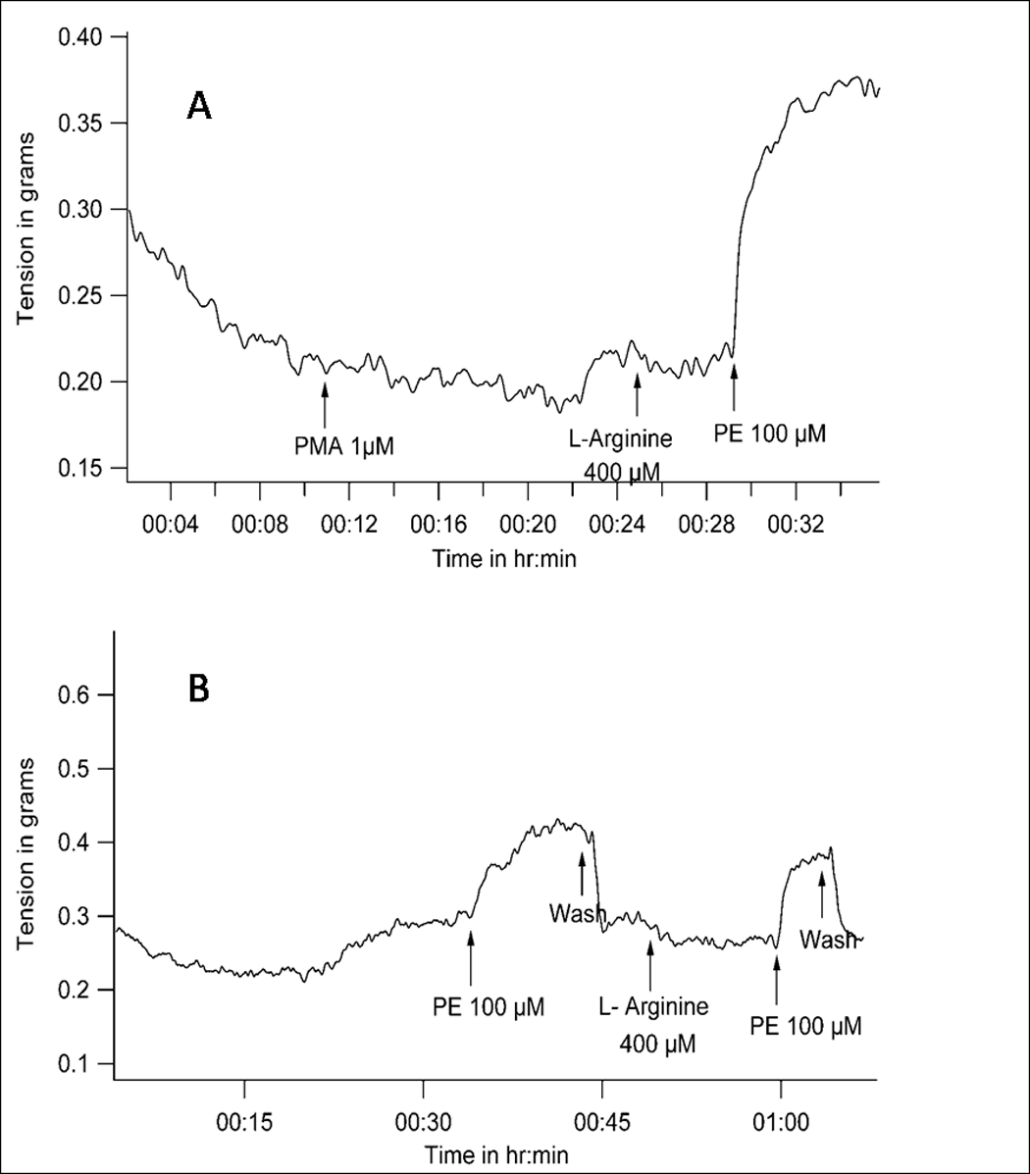
Representative tracings of tension recordings in spiral strips of small arteries from goat legs demonstrating that Phenylephrine administration leads to vasoconstriction only even in a high nitric oxide environment when protein kinase C is activated either with *(A)*, PMA or *(B)*, prior administration of Phenylephrine itself.

PKC was activated with PMA. PMA (1μmol/L) itself did not produce any change in vascular tone. After that L-Arginine too did not change tone. But Phenylephrine, subsequent to L-Arginine did not decrease tone in the presence of PMA. There was a robust vasoconstriction (Fig 7A) where the tone increased from 0.2 ± 0.13 gm to 0.41 ± 0.24 gm when PE was added in the environment described above (n = 5, P = 0.043 with WSR test).

Since alpha adrenergic stimulation itself activates PKC, it was investigated if prior use of PE itself can prevent reduction of tone due to the L-Arginine / PE combination. Fig 7B shows that this was the case. If the tissue is exposed to PE first, then PE caused only constriction afterwards, even in the presence of L-Arginine, with the tone going up from 0.36 ± 0.15 gm to 0.73 ± 0.61 gm (n = 5, P = 0.043 with WSR test).

### Summary of tension changes with the interventions employed

**Fig 8:**
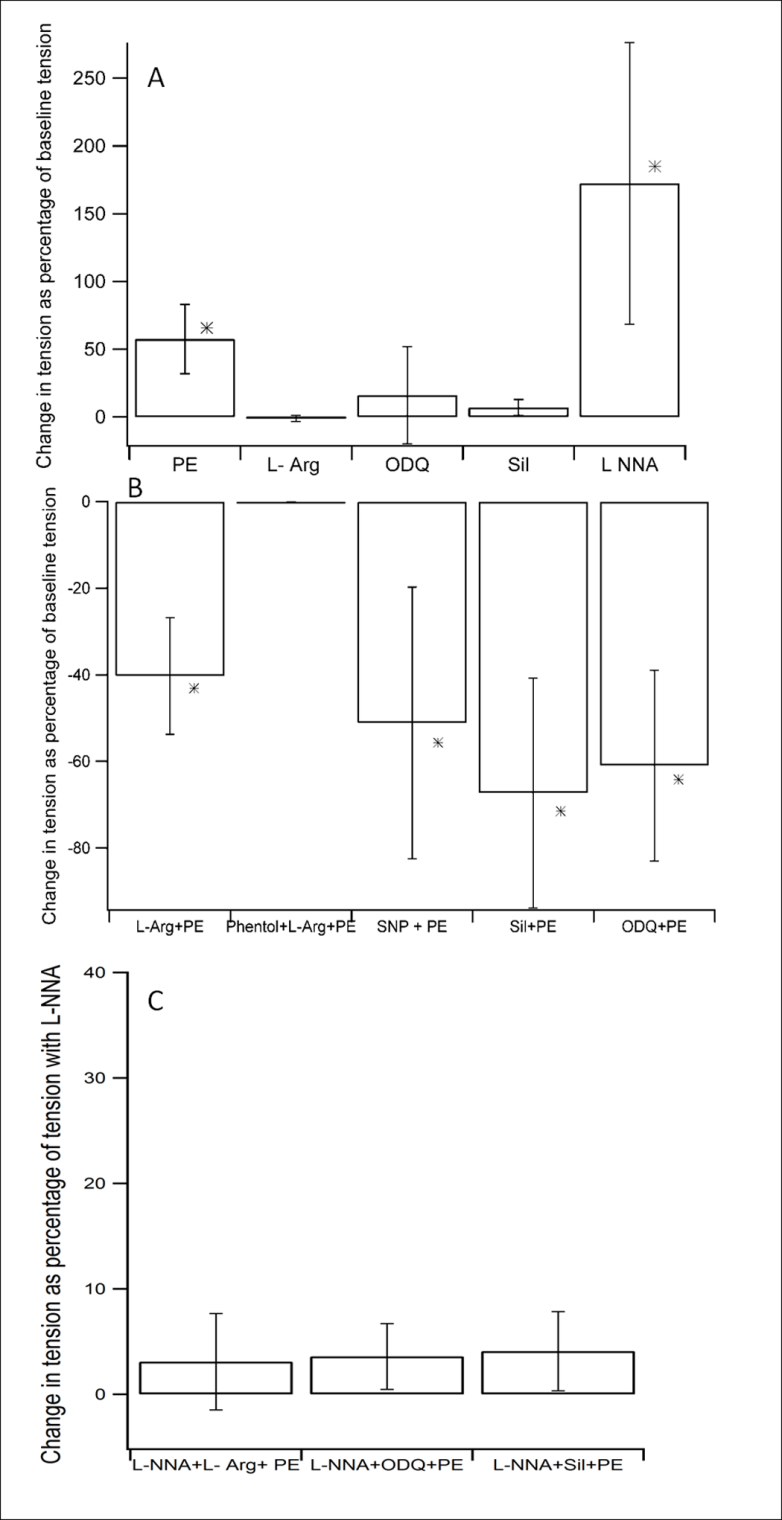
Bar diagrams (Mean ± S.D, _*_—P<0.*05*) representing percent changes in tension with each intervention, normalized to *(A& B)*, baseline tension *(C)*, tension with L-NNA alone.

The histograms (Fig 8A, 8B and 8C) summarize the changes in tone with the interventions that were employed.

### Histological examination and fluorescence staining

**Fig 9:**
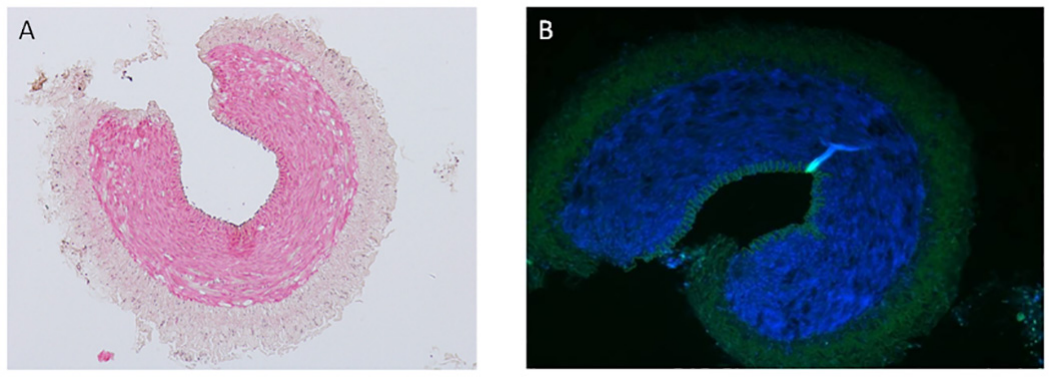
*(A)* The light microscopic view of eosin/hematoxylin stained, *(B)* the fluorescence microscopic view of a DAPI stained, spiral goat artery section showing that the endothelium is intact even after spiral sectioning.

To address the issue of whether the endothelium is intact and therefore capable of generating NO in the spiral sections, histological examination was performed. Light microscopic images of H & E stained sections of the spiral strip demonstrate that the endothelium is intact (Fig 9A). Fluorescence imaging of spiral strip also showed that the endothelium is intact (Fig 9B). The green fluorescence in the image is due to autofluorescence of collagen and elastin fibres [10]. DAPI stained nuclei of smooth muscle in tunica media appear blue.

## Discussion

PE is used as a vasoconstrictor and so are other adrenergic stimulants like adrenaline and noradrenaline. It is of considerable clinical significance, if alpha adrenergic agonists should lead to vasodilatation in some circumstances.

PE is an alpha-1 adrenergic agonist and its vasoconstrictor action occurs as outlined here. PE binding to the alpha adrenergic receptor results in the activation of a G protein which then activates a membrane enzyme called Phospholipase C (PLC). PLC converts Phosphoinositol bisphosphate (PIP_2_) in the membrane to Inositol triphosphate (IP_3_) and Diacyl glycerol (DAG). IP_3_ diffuses in the cytoplasm and mediates calcium release from sarcoplasmic reticulum (SR) by opening the IP_3_ receptors on the SR membrane. Calcium combines with calmodulin to activate the myosin light chain kinase which phosphorylates myosin light chain. Phosphorylated myosin light chain binds to actin to produce vasoconstriction. DAG can also activate PKC, which inhibits the enzyme myosin phosphatase, thereby preventing myosin dephosphorylation and consequent relaxation [1].

While PE and related alpha adrenergic agonists are employed in hypotensive settings to increase blood pressure, results presented here demonstrate that three different interventions, namely enhancement of NO levels (L-Arginine and SNP), sGC inhibitors (ODQ and methylene blue) and high cGMP levels (as occurs with Sildenafil) induce PE to reduce vascular tension, instead of causing vasoconstriction. Reduction of tension by PE in all these three circumstances is nitric-oxide dependent as L-NNA, a blocker of eNOS inhibits the response.

Nitric oxide is synthesised from L-Arginine by the enzyme Nitric Oxide Synthase (NOS). NOS can be broadly classified into constitutive NOS (cNOS) and inducible NOS (iNOS). The term cNOS includes endothelial NOS (eNOS) and neural NOS (nNOS) which are calcium-dependent constitutive enzymes. iNOS is not normally active. It is activated by certain pro-inflammatory agents like TNF, interleukins, endotoxins, interferon gamma (IFN) etc [11].

NO formed either from endothelium which has eNOS, or from cells which have iNOS, can diffuse into vascular smooth muscle to produce vasodilatation. One mechanism of NO-mediated vasodilatation involves activation of the enzyme sGC. sGC converts GTP to cGMP. cGMP activates PKG which in turn activates myosin light chain phosphatase, leading to vasodilatation. NO is stated to cause vasodilatation through cGMP independent mechanisms as well, by opening Ca activated K channel or by stimulation of Sarco/Endoplasmic reticulum calcium ATPase [12].

It is demonstrated here that, under control conditions, constitutive activity of eNOS and therefore a basal level of NO is important for maintaining a low basal tone in the goat arterial smooth muscle, as the eNOS inhibitor L-NNA leads to a robust increase in tone. However, such NO-mediated basal vasodilatation is not dependent on cGMP, as blockade of cGMP formation with ODQ or Methylene blue did not lead to an increase in tone. cGMP-independence of the basal state of vasodilatation is also proved by the fact that increasing cGMP levels with Sildenafil did not lead to a further decrease in basal tone.

PE under control conditions increases tone in the spiral strips of goat artery, which amounts to vasoconstriction. However, in 3 different circumstances, it causes decrease in vascular tone. One of these circumstances is where NO levels are higher, as in the case of L-Arginine or SNP. In the other two circumstances, NO levels are expected to be normal; and one of them involves low cGMP levels (sGC inhibition with ODQ or methylene blue), and the other involves high cGMP levels (cGMP phosphodiesterase inhibition with sildenafil). What is common to all these three scenarios is that the PE-induced reduction of tension in all these scenarios is NO-dependent and can be abolished by the eNOS inhibitor L-NNA. The action of PE in reducing vessel tension in the circumstances mentioned is not non-specific. Phentolamine, an alpha adrenergic blocker prevents reduction of tension in response to PE in the high NO environment, while propranolol, a beta blocker was unable to prevent the response. Therefore, alpha adrenergic activation is an important prerequisite to PE-induced reduction of vascular tension.

In the latter two scenarios, namely ‘sGC inhibition’ and ‘high cGMP levels’, NO levels *per se* are expected to be normal. To explain the NO-dependence of PE-induced tension reduction in these two scenarios, we postulate that basal NO is relieved of the need to bind to sGC (either directly as in the case of sGC inhibitors; or indirectly due to negative feed-back, when cGMP levels are high) and is diverted to a yet unidentified pathway wherein it couples with a down-stream second messenger of alpha adrenergic receptor activation to decrease vessel tension. This point of interaction of NO and PE could well be the enzyme PKC, as activation of PKC with either PMA or even PE at the start of the experiment, prevents further reduction in tension by the L-Arginine / PE combination. It is not as if excess NO alone inhibits PKC; it is important that there is simultaneous alpha receptor activation with PE, as both the NO sparing environment and PE are required to reduce vessel tension.

Basal levels of NO seem to perform two independent functions—one that involves activation of sGC and the other that maintains a low vascular tone by keeping the vessels in a relaxed state in a cGMP-independent manner. Any further reduction in tone by either higher level of NO or by sparing of even constitutive levels of NO (by obviating its need to bind to sGC) is strictly alpha-adrenergic activation-dependent. There is no additional reduction of vascular tone by excess NO if there is no alpha adrenergic receptor activation.

NO is implicated in various disease processes and sepsis is one of them. NO has a role in hypo-responsiveness of vasoconstrictors in septic shock as NO inhibitors reversed this hypo-responsiveness to catecholamine in septic rats [11]. In several experimental studies it has been shown that the plasma level of nitrite and nitrate are high during sepsis and the hypotension in sepsis can be prevented by inhibitors of NO synthesis.

It has been shown that norepinephrine administration can worsen the outcome in shock [12]. Prazosin has been reported to be useful in the treatment of cardiogenic shock [13]. A study done on rats reports that specific alpha 1 receptor blockade with prazosin may be useful in preventing decompensatory vasodilatation in hemorrhagic shock [14] and even improve the vasoconstrictor compensatory response [15]. More evidence needs to be generated on the usefulness of alpha adrenoceptor blockers in shock states, especially distributive shock.

## Abbreviations

PE: Phenylephrine
PKG: Protein kinase G
SNP: sodium nitroprusside

## References

[1] Graham RM, Perez DM, Hwa J, Piascik MT. alpha 1-adrenergic receptor subtypes. Molecular structure, function, and signaling. Circulation research. 1996;78(5):737–49. Epub 1996/05/01. . 8620593 10.1161/01.res.78.5.737

[2] Filippi S, Parenti A, Donnini S, Granger HJ, Fazzini A, Ledda F. alpha(1D)-adrenoceptors cause endothelium-dependent vasodilatation in the rat mesenteric vascular bed. The Journal of pharmacology and experimental therapeutics. 2001;296(3):869–75. Epub 2001/02/22. . 11181918

[3] Zschauer AO, Sielczak MW, Smith DA, Wanner A. Norepinephrine-induced contraction of isolated rabbit bronchial artery: role of alpha 1- and alpha 2-adrenoceptor activation. Journal of applied physiology (Bethesda, Md: 1985). 1997;82(6):1918–25. Epub 1997/06/01. . 9173959 10.1152/jappl.1997.82.6.1918

[4] Thorin E, Huang PL, Fishman MC, Bevan JA. Nitric oxide inhibits alpha2-adrenoceptor-mediated endothelium-dependent vasodilation. Circulation research. 1998;82(12):1323–9. Epub 1998/07/02. . 9648729 10.1161/01.res.82.12.1323

[5] Torp KD, Tschakovsky ME, Halliwill JR, Minson CT, Joyner MJ. beta-Receptor agonist activity of phenylephrine in the human forearm. Journal of applied physiology (Bethesda, Md: 1985). 2001;90(5):1855–9. Epub 2001/04/12. . 11299277 10.1152/jappl.2001.90.5.1855

[6] Joyner MJ, Dietz NM. Sympathetic vasodilation in human muscle. Acta Physiologica Scandinavica. 2003;177(3):329–36. 10.1046/j.1365-201X.2003.01090.x 12609003

[7] Bennett MA, Watt PA, Thurston H. Endothelium-dependent modulation of resistance vessel contraction: studies with NG-nitro-L-arginine methyl ester and NG-nitro-L-arginine. British journal of pharmacology. 1992;107(2):616–21. Epub 1992/10/01. 1422603 10.1111/j.1476-5381.1992.tb12792.x PMC1907882

[8] Van Hove CE, Van der Donckt C, Herman AG, Bult H, Fransen P. Vasodilator efficacy of nitric oxide depends on mechanisms of intracellular calcium mobilization in mouse aortic smooth muscle cells. British journal of pharmacology. 2009;158(3):920–30. Epub 2009/10/01. 10.1111/j.1476-5381.2009.00396.x 19788496 PMC2765610

[9] Goud C, DiPiero A, Lockette WE, Webb RC, Charpie JR. Cyclic GMP-independent mechanisms of nitric oxide-induced vasodilation. General pharmacology. 1999;32(1):51–5. Epub 1999/01/15. . 9888254 10.1016/s0306-3623(98)00059-7

[10] Richards-Kortum R, Sevick-Muraca E. Quantitative optical spectroscopy for tissue diagnosis. Annual review of physical chemistry. 1996;47:555–606. Epub 1996/01/01. 10.1146/annurev.physchem.47.1.555 . 8930102

[11] Hollenberg SM, Cunnion RE, Zimmerberg J. Nitric oxide synthase inhibition reverses arteriolar hyporesponsiveness to catecholamines in septic rats. The American journal of physiology. 1993;264(2 Pt 2):H660–3. Epub 1993/02/01. . 7680541 10.1152/ajpheart.1993.264.2.H660

[12] Povoa PR, Carneiro AH, Ribeiro OS, Pereira AC. Influence of vasopressor agent in septic shock mortality. Results from the Portuguese Community-Acquired Sepsis Study (SACiUCI study). Critical care medicine. 2009;37(2):410–6. Epub 2008/12/31. 10.1097/CCM.0b013e3181958b1c . 19114885

[13] Oliver LE, Horowitz JD, Dynon MK, Jarrott B, Brennan JB, Goble AJ, et al Use of dopamine and prazosin combined in the treatment of cardiogenic shock. The Medical journal of Australia. 1980;2 Suppl 1:42–5. Epub 1980/07/26. . 7421722 10.5694/j.1326-5377.1980.tb125819.x

[14] Bond RF, Johnson G 3rd. Cardiovascular adrenoreceptor function during compensatory and decompensatory hemorrhagic shock. Circulatory shock. 1984;12(1):9–24. Epub 1984/01/01. . 6323052

[15] Bond RF, Johnson G 3rd. Cardiovascular adrenoreceptor balance during hemorrhagic hypotension and shock. Circulatory shock. 1985;16(2):155–64. Epub 1985/01/01. . 3931932

